# Identification of zinc finger MIZ-type containing 2 as an oncoprotein enhancing NAD-dependent protein deacetylase sirtuin-1 deacetylase activity to regulate Wnt and Hippo pathways in non-small-cell lung cancer

**DOI:** 10.1186/s11658-024-00636-z

**Published:** 2024-09-12

**Authors:** Xueting Gan, Yuheng Feng, Yang Liu, Xuyong Lin, Xinmiao Yu, Xuezhu Rong, Qiang Han

**Affiliations:** 1 Department of Pathology, Shenbei New Area, College of Basic Medical Sciences and the First Hospital of China Medical University. No, 77 Puhe Road, Shenyang, 110122 Liaoning Province People’s Republic of China; 2 Department of Surgical Oncology and Breast Surgery, the First Hospital of China Medical University, Shenyang. No. 155 Nanjing North Street, Heping Area, Shenyang, 110001 Liaoning Province People’s Republic of China; 3 Department of Pathology, the First Hospital of China Medical University. No, 155 Nanjing North Street, Heping Area, Shenyang, 110001 Liaoning Province People’s Republic of China

**Keywords:** ZMIZ2, SIRT1, Non-small-cell lung cancer, Wnt and Hippo signaling pathway

## Abstract

**Background:**

Zinc finger MIZ-type containing 2 (ZMIZ2) can function as a coactivator and participate in the progression of certain malignant tumors; however, its expression and underlying molecular mechanism in non-small-cell lung cancer (NSCLC) remains unknown. In this study, we aim to analyze the expression of ZMIZ2 and its tumorigenic function in NSCLC, identifying its related factors.

**Methods:**

ZMIZ2 expression in NSCLC tissue samples and cell lines was examined using immunohistochemistry and western blotting; its biological role was investigated using in vivo and in vitro assays. The association between ZMIZ2 and NAD-dependent protein deacetylase sirtuin-1 (SIRT1) was demonstrated using mass spectrometry and immunoprecipitation experiments. Kyoto Encyclopedia of Genes and Genomes Pathway (KEGG)-based enrichment analysis, luciferase reporter assay, and real-time quantitative polymerase chain reaction (RT–qPCR) were conducted to verify the impact of ZMIZ2–SIRT1 combination on Hippo/Wnt pathways.

**Results:**

ZMIZ2 was highly expressed in NSCLC and positively associated with advanced pTNM staging, lymph node metastasis, and poor overall survival. Functional experiments revealed that ZMIZ2 promotes the proliferation, migration, and invasiveness of lung cancer cells—establishing its role as a promoter of oncogenes. Molecular mechanism studies identified SIRT1 as an assisted key factor interacting with ZMIZ2. KEGG enrichment analysis revealed that ZMIZ2 is closely related to Wnt/Hippo pathways; ZMIZ2–SIRT1 interaction enhanced SIRT1 deacetylase activity. Direct downregulation of intranuclear β-catenin and yes-associated protein (YAP) acetylation levels occurred independently of upstream proteins in Wnt/Hippo pathways; transcriptional activities of β-catenin-transcription factor 4 (TCF4) and YAP–TEA domain family transcription factors (TEADs) were amplified.

**Conclusions:**

ZMIZ2 promotes the malignant phenotype of lung cancer by regulating Wnt/Hippo pathways through SIRT1, providing an experimental basis for discovering novel biomarkers and developing tumor-targeted drugs.

**Graphical Abstract:**

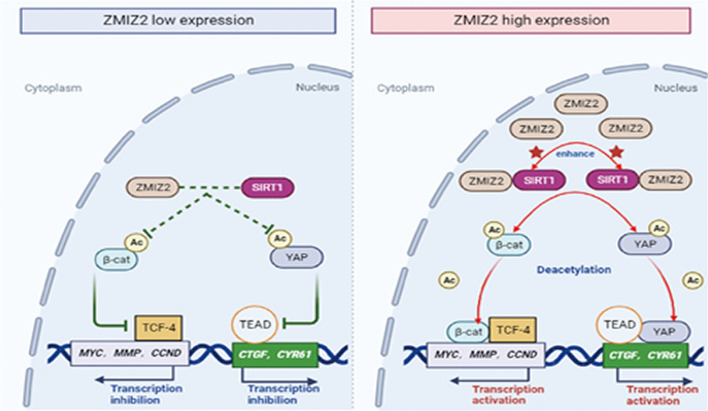

**Supplementary Information:**

The online version contains supplementary material available at 10.1186/s11658-024-00636-z.

## Background

The Wnt and Hippo pathways are evolutionarily conserved signaling pathways that play a crucial role in various physiological processes, such as embryonic development, cell proliferation, differentiation, and apoptosis. An imbalance in the activity of these pathways can lead to tumor formation, malignant progression, and drug resistance [1–5]. Previous studies, including our own, have identified numerous interactions and mutual influences between the Wnt and Hippo pathways; however, further investigations are necessary to detail the specific molecular crosstalk between these two pathways.

In the absence of Wnt signals in the classical Wnt pathway, the core factor β-catenin undergoes phosphorylation by a complex of adenomatous polyposis coli protein (APC) and two kinases—namely, casein kinase 1α (CK-1α) and glycogen synthase kinase-3β (GSK-3β)—leading to its degradation by proteasomes and maintenance at low cellular levels. Upon activation of Wnt signaling, the kinase activity in the degradation complex gets inhibited, thereby preventing β-catenin degradation; consequently, β-catenin accumulates in the nucleus. Within the nucleus, β-catenin binds to transcription factor 4 (TCF4), initiating the transcription of target genes—such as c-Myc protooncogene *(C-myc)*, G1/S-specific cyclin-D1 *(CCND1)* and matrix metallopeptidase-7 *(MMP7)* [6, 7]. The Hippo signaling pathway follows a comparable signaling process with an opposite effect. Upon activation of the Hippo pathway, the central kinase complex—comprising of mammalian sterile 20-like kinases 1/2 (MST1/2) and large tumor suppressor 1/2 (LATS1/LATS2)—undergoes a cascade of phosphorylation reactions, ultimately increasing phosphorylation of the effector protein, yes-associated protein (YAP). Phosphorylated YAP then undergoes degradation through the ubiquitin–proteasome pathway. However, when Hippo pathway activity is inhibited, dephosphorylated YAP gets translocated into the nucleus, where it binds to TEA domain family transcription factors (TEADs), initiating the transcription of its target genes—such as connective tissue growth factor *(CTGF)*, cysteine-rich angiogenic inducer 61 (*CYR61)*, and G1/S-specific cyclin-E (*CCNE)* [8, 9]. Despite these findings, the regulation of post-translational modifications of β-catenin and YAP and their impact on cotranscriptional activity within the nucleus remain unclear.

Zinc finger MIZ-type containing 2 (ZMIZ2), also known as hZIMP7, is an E3 SUMO-protein ligase PIAS-like protein inhibitor located on human chromosome 10, which has a molecular weight of approximately 115 kDa. The primary functional domains of this protein include the nuclear localization sequence (NLS), MIZ domain, and a proline-enriched region (Pro-Rich) situated at its C-terminus [10]. Existing literature indicates that ZMIZ2 primarily functions as a “coactivator” in various biological processes. For instance, ZMIZ2 interacts with SWI/SNF chromatin remodeling complexes, thereby contributing to chromatin remodeling processes and embryonic development of fruit flies, mice, and zebrafish [11, 12]. ZMIZ2 also interacts with androgen receptors, β-catenin, and ubiquitin-specific protease 6 (USP6), thereby enhancing their activity and promoting the malignant phenotype in tumors, such as prostate and colon cancers [13–15]. Recent studies have revealed that ZMIZ2 is highly expressed in triple-negative breast cancer, and hepatocellular carcinoma and is associated with poor prognosis [16, 17].

To the best of our knowledge, no experimental studies have directly explored the relationship between ZMIZ2 and lung cancer or unveiled the mechanisms underlying ZMIZ2 function in human lung cancer. Therefore, in this study, we investigated ZMIZ2 expression and its tumorigenic function in lung cancer. The primary mechanism underlying the tumorigenic effect of ZMIZ2 involves its binding with its “assisted helper”—NAD-dependent protein deacetylase sirtuin-1 (SIRT1)—which enhances the activity of SIRT1 deacetylase in the nucleus. This interaction leads to deacetylation of its substrates, β-catenin and YAP, resulting in an increased transcriptional activity of the β-catenin/TCF4 and YAP/TEAD complexes. Thus, this modulation affects the activity of both the Wnt and Hippo pathways.

## Methods

### Specimen collection and immunohistochemistry (IHC)

This study included a total of 93 lung cancer cases, 32 normal lung tissue samples for IHC (from 2018 to 2020), 32 pairs of fresh non-small-cell lung cancer tissues and their adjacent tissues for western blotting (2020), collected from the Department of Pathology, the First Hospital of China Medical University, 44 cases of adenocarcinoma, and 49 cases of squamous cell carcinoma, defined according to the 2021 edition of WHO lung cancer histological classification standards [18]; the average age of patients with lung cancer was 60 years. Using the UICC/AJCC TNM staging criteria (2023) [19], we categorized 61 cases as stages I and II and 32 cases as stage III cancer. Follow-up for all patients was considered from the date of surgery to the end of the follow-up period or the date of death attributed to recurrence or metastasis.

The polyclonal rabbit-derived ZMIZ2 antibody (HPA040716, 1:50; Sigma-Aldrich, St. Louis, MO) was used as the primary antibody, and tissue samples were incubated overnight at 4 ℃ with the antibody. Negative control was established using phosphate-buffered saline (PBS) instead of the primary antibody. Following this, the samples were incubated at 37 ℃ for 30 min with a biotin-labeled secondary antibody (MaiXin, Fuzhou, China), and DAB staining was performed.

Five randomly selected fields of view were assessed for each tissue slice, with a count of 100 cells per field under an optical microscope (Nikon, Japan). ZMIZ2 expression levels were categorized into five stages based on the percentage of stained cells: 0 (no staining), 1 (1–25%), 2 (26–50%), 3 (51–75%), and 4 (> 76%). Additionally, based on the intensity of cell staining, ZMIZ2 expression was further classified into three levels: 0 (no staining), 1 (light yellow particles), and 2 (deep yellow or yellowish-brown particles). Each tissue slice received both percentage and coloring scores, which were multiplied to obtain the final score. Because the staining scores in most normal lung bronchial and alveolus epithelia were less than 4, a score ≥ 4 was considered a positive expression, whereas a score < 4 was considered a negative expression.

### Non-small-cell lung cancer (NSCLC) cell lines and culture

The human immortalized bronchial epithelial cell line (HBE, no. AC338600) and lung adenocarcinoma cell line (SPC-A-1, no. XY-XB-1057) were obtained from ATCC Cell Library (Manassas, VA). The LK2 cell line was received from Doctor Hiroshi Kijima (Department of Pathology and Bioscience, Hirosaki University Graduate School of Medicine, Japan). NCI-A549 (no. TCHu150), NCI-H1299 (no. SCSP-589), Calu-1 (no. TCHu192), NCI-H661 (no. SCSP-5071), and NCI-H460 (no. SCSP-584) cells were purchased from the Cell Bank of the Chinese Academy of Sciences (Shanghai, China). All cell lines were subjected to short tandem repeat (STR) analysis and mycoplasma testing. Calu-1 cells were cultured in Myco5A medium, and the remaining six lung cancer cell lines were cultured in RPMI-1640 medium. All culture media contained 10% calf serum (Invitrogen, Carlsbad, CA) and 100 U/mL penicillin (Sigma-Aldrich). The cell lines were cultured in a 5% CO_2_ incubator.

### ZMIZ2 overexpression plasmid, CRISPR-CAS9-sgRNA lentivirus, and cell transfection

Myc-tagged pCMV6 empty vector and pCMV6-ZMIZ2 plasmids were purchased from OriGene (no. PS-100001 and no. RC-216992, respectively; Rockville, MD). The pcDNA3.1 empty vector (no. 52535), pcDNA3.1-FLAG-SIRT1 (no. 1791), pcDNA3.1-FLAG-SIRT1-H363Y (no. 1792), GFP- β-catenin (no. 71367), pGL3b 8xGTIIC luciferase (no. 34615), and Super 8 × Topflash (no. 12456) plasmids were purchased from Addgene (Cambridge, MA). The pRL-TK vector (no. E2241) was purchased from Promega (Madison, Wisconsin). HA-TCF4 (NM_001083962), HA-TEAD (NM_021961), pEGFP-N1 empty vector, pEGFP-N1-YAP (NM_001130145), and Myc-ZMIZ2 (NM_001300959) mutant plasmids (ZMIZ2-ΔNLS, ZMIZ2-ΔMIZ, and ZMIZ2-ΔPro-rich) were constructed by Baihao Company (Shenyang, China). Control siRNA (sc-37007), siRNA-ZMIZ2 (sc-89373), and siRNA-SIRT1 (sc-40986) were purchased from Santa Cruz Technology Inc. (CA). Lentivirus-ZMIZ2 and sgRNA-ZMIZ2 lentivirus plasmids were purchased from GeneChem (Shanghai, China). Cells were transiently transfected using Lipofectamine 3000 (Invitrogen, Carlsbad, CA) following the manufacturer’s instructions. In case of stable transfection, purinomycin (Sigma-Aldrich) was employed during clone screening.

### Protein extraction and western blotting

Assays were performed as described previously [6]. ZMIZ2 (sc-163547, IB/1:200, IP/1:50), GFP-Tag (sc-9996, IB/1:500, IP/1:50), TCF4 (sc-166699, IB/1:200), and GAPDH (sc-293335, IB/1:1000) were purchased from Santa Cruz Biotechnology. SIRT1 (no. 8469, IB/1:1000, IP/1:50), Myc Tag (no. 2276, IB/1:1000, IP/1:50), FLAG-Tag (no. 14793, IB/1:1000), HA Tag (no. 3724, IB/1:1000), C-myc (no. 9402, IB/1:1000), MMP7 (no. 3801, IB/1:1000), CyclinD1 (no. 2922, IB/1:1000), CTGF (no. 86641, IB/1:1000), CYR61 (no. 14479, IB/1:1000), MST (no. 3682 IB/1:1000), p-MST (no. 49332, IB/1:1000), p-LATS1 (no. 8654, IB/1:1000) LATS1 (no. 3477, IB/1:1000), YAP (no. 14074, IB/1:1000, IP/1:50), action (no. 3700, IB/11000), MOB1 (no. 13730, IB/1:1000), SAV1 (no. 13001, IB/1:1000), β-catenin (no. 8480, IB/1:1000, IP/1:50), acetyl-β-catenin (Lys49, no. 9534, IB/1:1000), acetyl lysine antibody (no. 9441, IB/1:1000), LaminB1 (no. 13435, IB/1:1000), and Tublin (no. 2148, IB/1:1000) were all purchased from Cell Signalling Technology (Danvers, MA). After incubation with peroxidase-coupled anti-mouse IgG and anti-rabbit IgG (Santa Cruz Biotechnology) antibodies at 37 °C for 1 h, bound proteins were viewed using ECL (Thermo Fisher Scientific, Waltham, MA) and detected using Bio-Rad Systems (California, USA). ImageJ software (version 18.0) was used to measure grayscale values on strips [20], and the relative expression level was determined by normalizing to GAPDH. Human recombination protein Wnt3a (no. 5036-WN, R&D Systems, France) was dissolved in PBS containing 0.2% BSA to achieve a concentration of 10 μg/mL and was utilized in the assays at a final concentration of 50 ng/mL. The experiments were repeated three times, and the mean was subsequently calculated.

### RNA extraction and real-time quantitative polymerase chain reaction (RT–qPCR)

Total RNA was extracted from cells using RNeasy Plus Mini Kit (Qiagen, Hilden, Germany), and RT–qPCR was performed using SYBR Green PCR Master Mix (Applied Biosystems, Foster City, CA) with 20 μL sample in a 7900HT Fast real-time quantitative PCR system (Applied Biosystems, Foster City, CA). The PCR conditions included an initial step at 50 ℃ for 2 min, followed by 95 ℃ for 10 min, and then a cycling step at 95 ℃ for 40 s and 60 ℃ for 60 s. GAPDH was used as an internal reference, with the relative expression level of the gene represented as ΔCt (Ct _value of the gene_—Ct _value of the internal reference_)_._ The changes in the relative expression of genes were calculated as 2^–ΔΔCt^ using the Ct method [21], and all experiments were conducted in triplicate. Table 1 presents the primer sequences used in RT–qPCR analysis.

**Table 1:** Primers for real-time reverse transcriptase polymerase chain reaction

Primer sequences (5′ → 3′)	
*ZMIZ2*	5′-TCCACTGACTTCACGCAAGC-3′
	5′-TATGCCAGTAGGGTTCATGCC-3′
*C-MYC*	5′-GGCTCCTGGCAAAAGGTCA-3′
	5′-CTGCGTAGTTGTGCTGATGT-3′
*MMP7*	5′-TCGGAGGAGATGCTCACTTCGA-3′
	5′-GGATCAGAGGAATGTCCCATACC-3′
*CCND1*	5′-GCTGCGAAGTGGAAACCATC-3′
	5′-CCTCCTTCTGCACACATTTGAA-3′
*CTGF*	5′- AACTGCAACCTCTCGCACTG-3′
	5′- GCTCGGGCTCCTTGTAATTCT-3′
*CYR61*	5′- CTCGCCTTAGTCGTCACCC-3′
	5′- CGCCGAAGTTGCATTCCAG-3′
*CCNE*	5′-GCCAGCCTTGGGACAATAATG-3′
	5′-CTTGCACGTTGAGTTTGGGT-3′
*GAPDH*	5′- GGAGCGAGATCCCTCCAAAAT-3′
	5′- GGCTGTTGTCATACTTCTCATGG-3′

*ZMIZ2* Zinc finger MIZ-type containing 2, *C-MYC* c-MYC proto-oncogene, *MMP7* Matrix metallopeptidase-7, *CCND1* G1/S-specific cyclin-D1, *CTGF* Connective tissue growth factor, *CYR61* Cysteine-rich angiogenic inducer 61, *CCNE* G1/S-specific cyclin-E, *GAPDH* Glyceraldehyde-3-phosphate dehydrogenase

### Dual-luciferase reporter assay

YAP/TEAD transcriptional activity was measured using a luciferase assay based on the pGL3b_8xGTIIC-luciferase plasmid purchased from Addgene (plasmid no. 34615); β-catenin/TCF4 transcriptional activity was measured using Super 8xTopflash plasmid purchased from Addgene (plasmid no. 12456). Cells were transfected to express the indicated proteins, and Renilla luciferase was used as a control for signal normalization. Dual luciferase assays were performed according to the manufacturer’s protocol (Progema, WI). Three independent transfections were performed for each experiment. Data were normalized to the empty vector control and presented as mean ± SD.

### Nuclear-cytoplasmic protein separation

The cells were trypsinized and washed with cold PBS, and the cell pellets were resuspended in an ice-cold lysis buffer supplemented with protease inhibitors (210 mM mannitol, 70 mM sucrose, 5 mM Tris, pH 7.5, and 1 mM EDTA). After 15 min of incubation on ice, the cells were homogenized. The nuclei were isolated via centrifugation (12,000 rpm, 10 min, 4 °C), and the nuclear pellets were washed with PBS and resuspended on ice for 5 min in RIPA buffer (Santa Cruz Biotechnology, CA). Following centrifugation and sedimentation, the supernatant (nuclear fraction) was collected and transferred to a new 1.5-mL tube. The lysate was quantified using the Bradford assay, and an equivalent amount of total protein was subjected to western blotting.

### Mass spectrometry analysis, immunoprecipitation, and Kyoto encyclopaedia of genes and genomes (KEGG)

For the immunoprecipitation assay, whole-cell extracts were prepared after transfection and incubated overnight with indicated antibodies and Protein A/G beads (no. sc-2003, Santa Cruz Technology). Beads were then washed three times with lysis buffer, and immunoprecipitates were eluted for detection using mass spectrometry. The results obtained from mass spectrometry were integrated and statistically analyzed, subsequently incorporating KEGG signaling pathway enrichment analysis through OmicShare Tools (omicshare.com/tool/). The BioGird online protein–protein interaction database (thebiogrid.org) was applied to predict the underlying interacted proteins with ZMIZ2.

### Cellular immunofluorescence staining

Cells were fixed using 4% paraformaldehyde and sealed with 3% BSA for 1 h. ZMIZ2 rabbit polyclonal antibody (HPA040716, 1:50; Sigma Aldrich), β-catenin (1:100; Cell Signalling Technology), and YAP (no. 14074, 1:100) antibodies were incubated with the cells overnight at 4 ℃. Subsequently, the cells were incubated with a goat anti-rabbit/mouse secondary antibody (1:5000; Cell Signalling Technology). Nuclei were stained with DAPI and observed via confocal scanning using a Radiance 2000 laser confocal microscope (Carl Zeiss, Thornwood, NY).

### Colony formation, matrix adhesive invasion, and MTT assays

Colony formation assay: Following a 48-h period post overexpression or interference in the tumor cell transfection experiment, the cells were seeded into a 6-cm cell culture dish (1000 cells/dish) and incubated for 12 days. Subsequently, the dish was washed with PBS and stained with haematoxylin. Colonies with > 50 cells were then counted.

MTT assay: Approximately 3000 transfected-NCI-H1299 and Calu-1 cells were cultured in a 96-well plate with 10% serum for 24 h. Subsequently, 20 μL of a 5 mg/ml MTT (thiazolyl blue) solution was added to each well, followed by incubation at 37 ℃ for 4 h. After removing the solution, MTT crystals were dissolved in 150 mL of dimethyl sulfoxide (DMSO), and absorbance was measured at 490 nm using a spectrophotometer (Bio-Rad, CA).

Cell matrix adhesive invasion assay: The matrix adhesive (BD Biosciences, CA) was diluted in Dulbecco’s modified Eagle medium (DMEM) medium (1:3 ratio) in a 24-well plate, excluding any additives, and seeded in an 8-μm pore size upper chamber (Costa, Shanghai, China). After 48 h of transfection, the tumor cells (5 × 10^5^ cells) were placed in the upper chamber and incubated for 16 h. A culture medium containing 10% calf serum was placed in the lower chamber. After 24 h of cultivation post-seeding, the cells were fixed in ice-cold methanol for 15 min and stained with haematoxylin. Ten randomly selected fields of view were used to count the number of cells invading the subventricular space using a Nikon optical microscope (Japan). The experiment was repeated three times, and the average value was calculated.

### Animal experiments

Animal experiments were performed in accordance with the ethical regulations governing animal studies at the China Medical University (no. CMU20231358). The maximal tumor size permitted by the ethics committee/ was 2000 mm^3^, and the maximal tumor size in our study was not exceeded. Female BALB/c nude mice (*n* = 25), aged 4 weeks and weighing 16–20 g, were purchased from the Charles River Company (Beijing, China). Both the feed and drinking water were sterilized in a semibarrier system maintained at constant temperature and humidity. The cell concentration in each group (0.2 mL) was adjusted to 5 × 10^6^ pieces/mL and subcutaneously injected into the dorsal regions of the nude mice. Following continuous observation for 5 weeks postinjection day, the mice were euthanized, and the weight and volume of the subcutaneously transplanted tumors were recorded. Tumor volume was calculated using the equation: length × width^2^ /2.

### Ethics statement

The study design was approved by the Institutional Review Board of China Medical University (no. LS [2019] 003). All participants signed the informed consent form, and the study was conducted in accordance with the principles of Declaration of Helsinki. The animals used in this study were treated according to the National Institutes of Health Guide for the Care and Use of Laboratory Animals (NIH Publications no. 8023, revised 1978).

### Statistical analysis

All data were statistically analyzed using SPSS version 27.0 (Chicago, IL). The chi-square test was employed to examine the association between ZMIZ2 expression and clinical pathological factors. Kaplan–Meier analysis was used to assess the relationship between ZMIZ2 and the overall prognosis of patients with lung cancer. The differences between groups were analyzed using a *t*-test or two-way analysis of variance (ANOVA). A significance level of *P* < 0.05 was considered statistically significant.

### Role of the funding source

The funders (National Natural Science Foundation of China) had no role in the study design, sample collection and examination, animal experiments, data analysis and interpretation, or writing of the manuscript. The corresponding authors had full access to all the data and final responsibility for the decision to submit for publication.

## Results

### ZMIZ2 is highly expressed in NSCLC and associated with poor overall survival

**Fig. 1:**
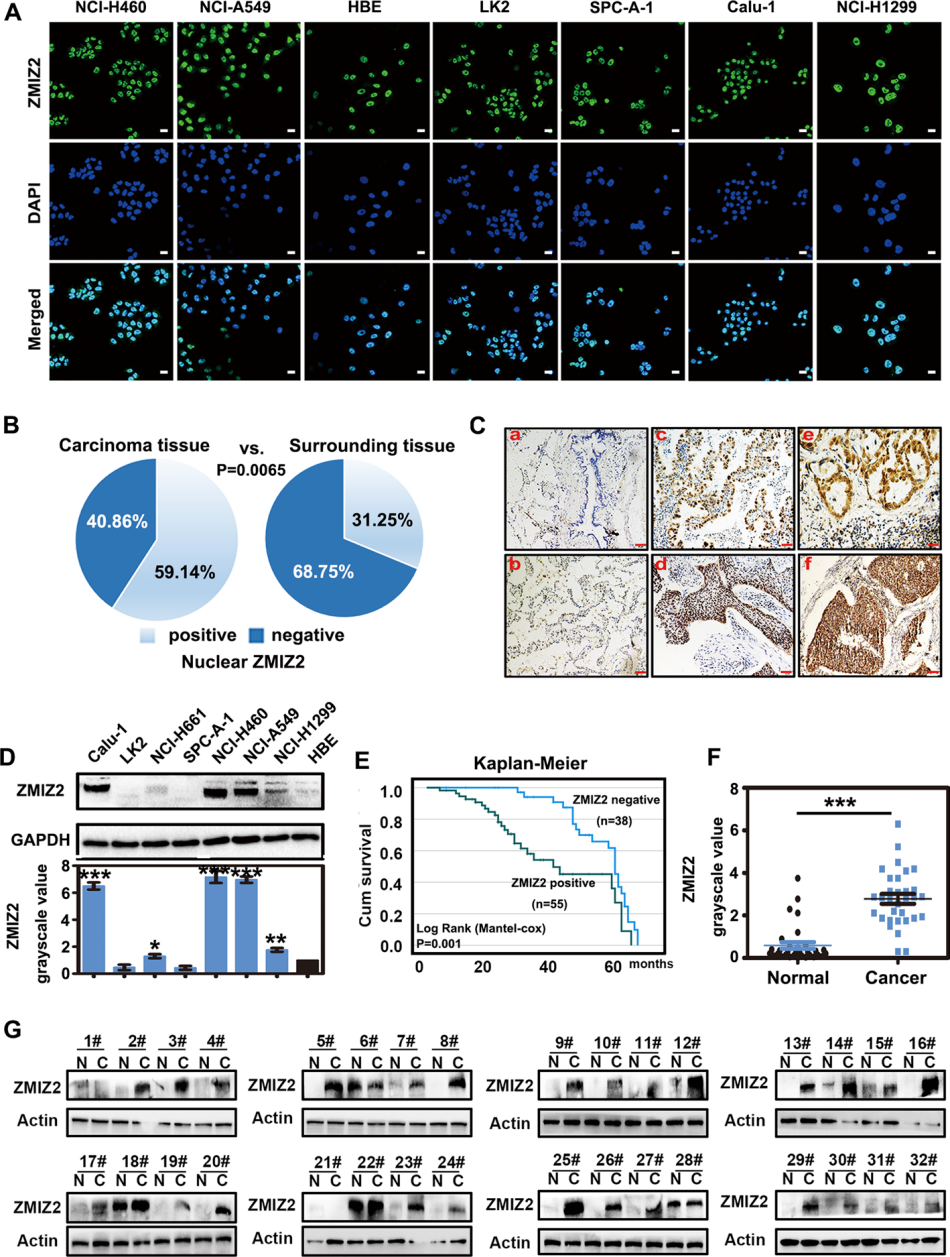
ZMIZ2 expression in NSCLC cells and specimens and its association with poor overall survival. **A** In a panel of NSCLC cell lines (*n* = 7) and an immortal bronchial epithelial cell line (HBE), immunofluorescence assays revealed nuclear localization of ZMIZ2. **B**, **C** IHC analysis demonstrated weak or negative expression of ZMIZ2 in human bronchial epithelium and alveolar epithelium (10/32, a, b). Positive expression was observed in the nucleus in adenocarcinoma and squamous cell carcinoma tissues (55/93, c, d). In lymph node metastatic lung adenocarcinoma and squamous cell carcinoma, ZMIZ2 exhibited stronger nuclear expression (38.2% versus 82.0%, *P* < 0.05, e, f) (magnification, 400× ; scale bar, 50 μm). **D** Western blotting analysis indicated higher ZMIZ2 expression in tumor cell lines (5/7) than that in HBE. Columns represent mean values, and the bars represent the standard deviation (SD). (*P < 0.05, ** P < 0.01, *** P < 0.001). Grayscale values of ZMIZ2 were measured by ImageJ from triplicate experiments. **E** Kaplan–Meier analysis indicated a significantly lower 5 year overall survival rate for patients with positive ZMIZ2 expression (*n* = 55, 58.0 ± 1.2 months) compared with those with negative ZMIZ2 expression (*n* = 38, 39.0 ± 10.4 months). **(***P* = 0.001**)**. **F**, **G** Scatter plot analysis detected using western blotting showed higher ZMIZ2 expression in cancer tissues (26/32) than that in the adjacent normal lung tissues (***P < 0.001)

To explore the expression and biological role of ZMIZ2 in NSCLC, we performed immunofluorescence, IHC, and western blotting analyses using established lung cancer cell lines and clinical samples. As a result, the following parameters were observed: (1) localization: ZMIZ2 was localized within the nuclei of human immortalized bronchial epithelial cells and lung cancer cells; (2) expression profile: ZMIZ2 was expressed at low levels in bronchial and alveolar epithelial cells of normal lung tissue (10/32), but was highly expressed in multiple lung cancer cell lines (5/7) and observed in over 59.14% (55/93) of NSCLC cases. The difference in expression between para-cancerous and cancerous tissues was significant (Fig. 1A–D, P = 0.0065). Elevated nuclear ZMIZ2 expression was significantly associated with advanced p-TNM staging (*P* = 0.028), lymph node metastasis (*P* = 0.016), and poor overall survival (*P* = 0.001) in NSCLC (Fig. 1E, Table 2). Additionally, western blotting analysis was performed on 32 pairs of fresh lung cancer tissues and their adjacent tissues, revealing that protein expression of ZMIZ2 in cancer tissues was significantly higher than that in the adjacent tissues (24/32) (Fig. 1F, G, P < 0.0001). These abovementioned results suggest that ZMIZ2 may function as an oncoprotein in NSCLC.

**Table 2:** Association of ZMIZ2 expression with clinical and pathological characteristics in NSCLC

Clinicopathological	N	Positive	Negative	*χ*^2^	P
Characteristics					
Age (years)					
< 60	31	18	13	0.022	1.000
≥ 60	62	37	25		
Sex					
Male	54	30	24	0.685	0.522
Female	39	25	14		
Histological type					
Squamous cell carcinoma	44	22	22	2.887	0.097
Adenocarcinoma	49	33	16		
Differentiation					
Well	33	23	10	2.359	0.186
Moderate and poor	60	32	28		
TNM classification					
I + II	61	31	30	5.079	0.028*
III	32	24	8		
Lymph node metastasis					
Negative	34	15	19	6.032	0.016*
Positive	59	40	19		

* statistically significant (*P*<0.05)

### ZMIZ2 promotes the malignant phenotype of NSCLC cells

**Fig. 2:**
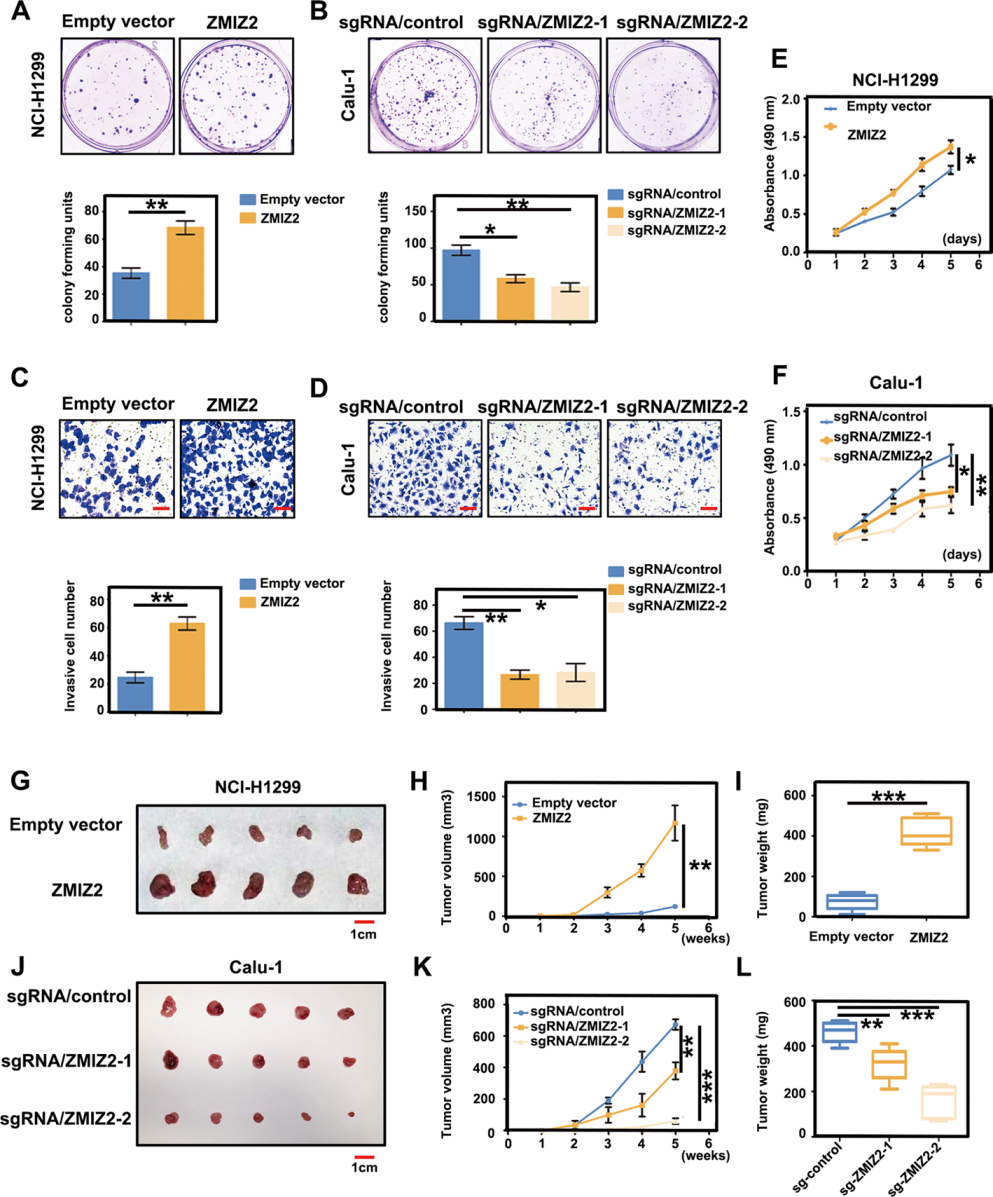
Functions of ZMIZ2 as an oncogene promoting the malignant phenotype of NSCLC. **A**, **B** ZMIZ2 enhances the clonogenic potential of NSCLC cells. Lentivirus particles carrying ZMIZ2 plasmids were used for stable overexpression, while sgRNA-ZMIZ2 was used for knockdown in NCI-H1299 and Calu-1 cells, respectively. Changes in proliferative ability were assessed through colony formation experiments. **C**, **D** ZMIZ2 overexpression increased the invasive ability of NCI-H1299 cells in matrix gel invasion assays. In contrast, Calu-1 cells with ZMIZ2 knockdown exhibited a significant reduction in invasive potential compared with the control group. **E**, **F** MTT assay revealed that ZMIZ2 overexpression enhanced the proliferative ability of NCI-H1299 cells, while knockout yielded the opposite result. **G**–**L** Following stable ZMIZ2 overexpression in NCI-H1299 cells, the transplanted tumors in nude mice (*n* = 5) exhibited significantly greater volume and weight than those in the control group (*n* = 5) (**G**–**I**). In contrast, ZMIZ2 knockdown in Calu-1 cells significantly increased the volume and mass of the transplanted tumors (*n* = 5) (**J**–**L**). Data represent the average of three independent experiments. Columns represent mean values, and the bars represent the standard deviation (SD). (*P < 0.05; **P < 0.01; ***P < 0.001)

To further explore the impact of ZMIZ2 on malignant phenotypes, such as invasion and proliferation in lung cancer, we manipulated ZMIZ2 expression in NCI-H1299 (relatively low ZMIZ2 expression) and Calu-1 (high ZMIZ2 expression) cell lines. Lentivirus-coated ZMIZ2 plasmids and sgRNA-ZMIZ2 were used to induce overexpression and knockdown ZMIZ2 expression (purinomycin screening). Subsequent colony assays, transwell, and MTT assays revealed that ZMIZ2 overexpression enhances the colonic (NCI-H1299, empty vector versus ZMIZ2: 35 ± 4 versus 68 ± 5, *P* = 0.0059), invasive (NCI-H1299, empty vector versus ZMIZ2: 25 ± 4 versus 63 ± 5, *P* = 0.0030), and proliferative capabilities (NCI-H1299, empty vector versus ZMIZ2: 1.072 ± 0.054 versus 1.372 ± 0.084, *P* = 0.0173) of tumor cells (Fig. 2A, C, E). Conversely, ZMIZ2 knockdown significantly inhibited malignant phenotype of the tumor (Calu-1: colony assay: sg-control versus sg-ZMIZ2-1: 97 ± 7 versus 58 ± 5, *P* = 0.0118; sg-control versus sg-ZMIZ2-2: 97 ± 7 versus 46 ± 6, *P* = 0.0054; transwell assay: sg-control versus sg-ZMIZ2-1: 66 ± 5 versus 26 ± 4, *P* = 0.0028; sg-control versus sg-ZMIZ2-2: 66 ± 5 versus 28 ± 7, *P* = 0.0109; MTT assay: sg-control versus sg-ZMIZ2-1: 1.089 ± 0.099 versus 0.748 ± 0.039, *P* = 0.0130; sg-control versus sg-ZMIZ2-2: 1.089 ± 0.099 versus 0.620 ± 0.074, *P* = 0.0056) (Fig. 2B, D, F). In vivo experiments involving subcutaneous tumor formation in nude mice revealed that, compared with those in the control group, the volume and weight of subcutaneously transplanted tumors were significantly increased in mice with ZMIZ2 ectopic expression (NCI-H1299, volume: empty vector versus ZMIZ2: 122.4 ± 19.8 mm^3^ versus 1168.0 ± 220.8 mm^3^, *P* = 0.0015; weight: 74.2 ± 17.9 mg versus 420.0 ± 31.7 mg, *P* < 0.0001) (Fig. 2G–I). Conversely, the group with ZMIZ2 knockdown exhibited opposite results (Calu-1: volume: sg-control versus sg-ZMIZ2-1: 673.0 ± 33.6 mm^3^ versus 373.8 ± 54.4 mm^3^, *P* = 0.0017; sg-control versus sg-ZMIZ2-2: 673.0 ± 33.6 mm^3^ versus 59.8 ± 18.0 mm^3^, *P* < 0.0001; weight: sg-control versus sg-ZMIZ2-1: 462.0 ± 20.6 mg versus 320.0 ± 32.3 mg, *P* = 0.0059; sg-control versus sg-ZMIZ2-2: 462.0 ± 20.6 mg versus 158.0 ± 32.7 mg, *P* < 0.0001) (Fig. 2J–L). Overall, the evidence from in vitro and in vivo experiments demonstrates that ZMIZ2 functions as an oncogene in NSCLC, enhancing its biological role and promoting malignant phenotype of tumor cells.

### Identification of SIRT1 as an assisted partner of ZMIZ2 coactivator

**Fig. 3:**
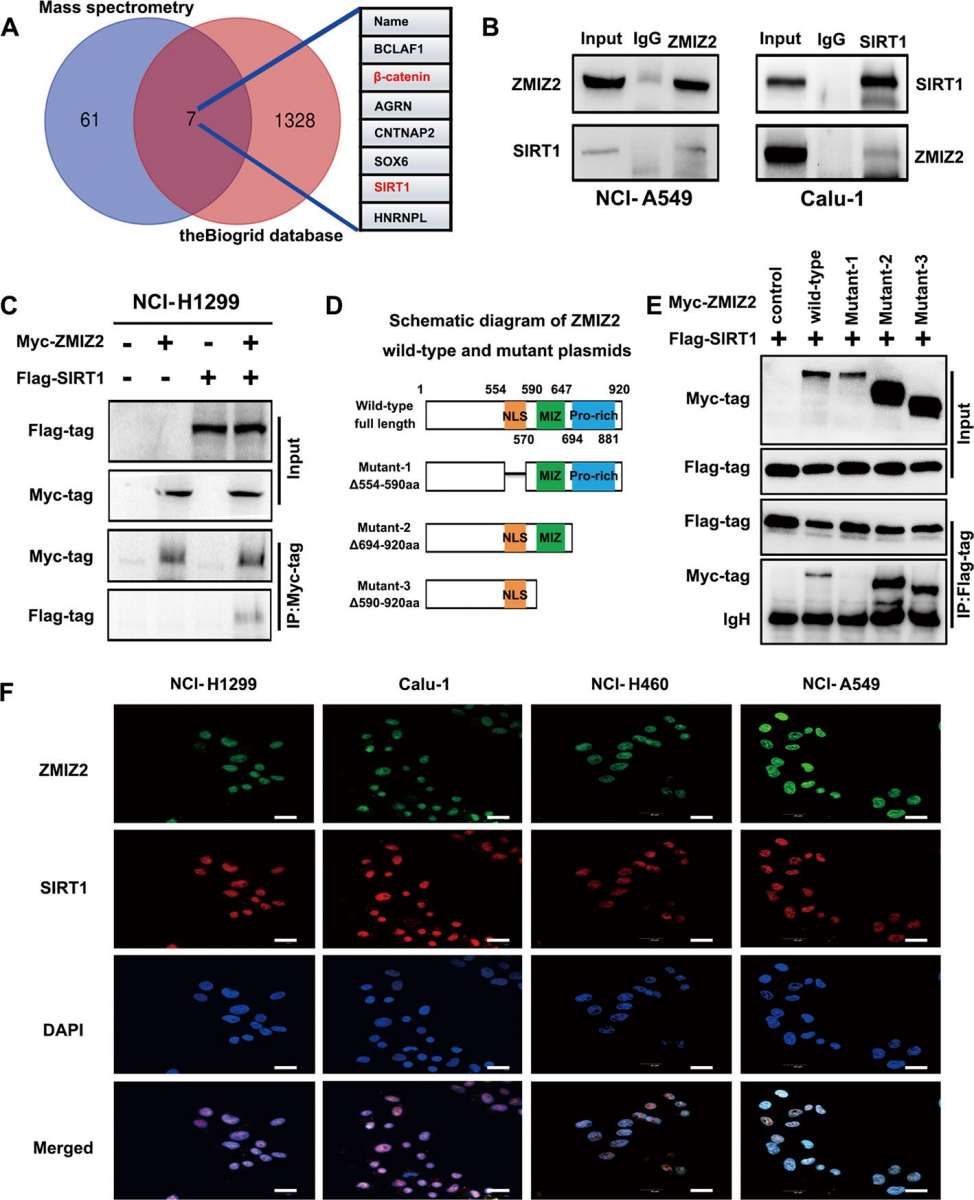
Identification of SIRT1 as an “assisted partner” of ZMIZ2 coactivator. **A** Proteins interacting with ZMIZ2 were identified through mass spectrometry analysis and a protein interaction network database (the Biogrid database). **B** Interaction between ZMIZ2 and SIRT1. Cell lysates from NCI-A549 and Calu-1 cells were subjected to immunoprecipitation with anti-ZMIZ2, anti-SIRT1, or control IgG, respectively, and precipitates were analyzed using western blotting. **C** Cotransfection of Myc-ZMIZ2 and Flag-SIRT1 plasmids in NCI-H1299, followed by immunoprecipitation, validated the interaction between ZMIZ2 and SIRT1 at the exogenous level. **D** Schematic diagram illustrating ZMIZ2 wild-type and mutant plasmids. **E** Immunoprecipitation after cotransfection of Flag-SIRT1, Myc-ZMIZ2, and corresponding mutants in NCI-H1299 revealed that mutants lacking NLS sequences were incapable of interacting with SIRT1. *NLS* nuclear location sequence, *IgH* IgG heavy chain. **F** Cellular immunofluorescence assay was conducted to observe the colocalization of ZMIZ2 and SIRT1 in the nuclei of multiple lung cancer cell lines (magnification, 400× ; scale bar: 50 μm)

Studies suggests the coactivator function of ZMIZ2; however, it remains unclear whether the functional proteins it may assist in promoting NSCLC, beyond those previously reported, may also contribute to biological functions mediated by other factors. This aspect was crucial to our investigation. Consequently, we first transfected the Myc-ZMIZ2 plasmid into the NCI-H1299 cell line and then used Myc-tag monoclonal antibodies to collect protein complexes for subsequent mass spectrometry analysis. After removing nonspecific binding proteins and those that were not clearly annotated, we obtained a set of 1328 genes that could potentially bind to ZMIZ2 (Supplementary Table 1). In addition, to further narrow this range, we searched for genes that can bind to ZMIZ2 in the protein interaction database BioGrid.com and obtained a dataset containing 61 genes. Using the intersection of the two datasets, we ultimately obtained seven genes that can bind to ZMIZ2. The results revealed that, in addition to the previously reported interaction with β-catenin [14], ZMIZ2 also binds to the deacetylase SIRT1, which is closely associated with tumor progression (Fig. 3A). Endogenous immune coprecipitation assays performed in NCI-A549 and Calu-1 cells confirmed the ZMIZ2–SIRT1 interaction (Fig. 3B). Furthermore, cotransfection of ZMIZ2 and SIRT1 plasmids into NCI-H1299 cells supported this interaction through exogenous immunoprecipitation experiments (Fig. 3C). Immunoprecipitation experiments demonstrated that ZMIZ2, when lacking the NLS domain, could not interact with SIRT1 (Fig. 3D, E). In addition, laser confocal microscopy experiments showed that ZMIZ2 and SIRT1 colocalized within the tumor nucleus across various lung cancer cell lines (Fig. 3F).

### ZMIZ2 functions as a positive regulator of Wnt and negative regulator of Hippo signaling pathway

**Fig. 4:**
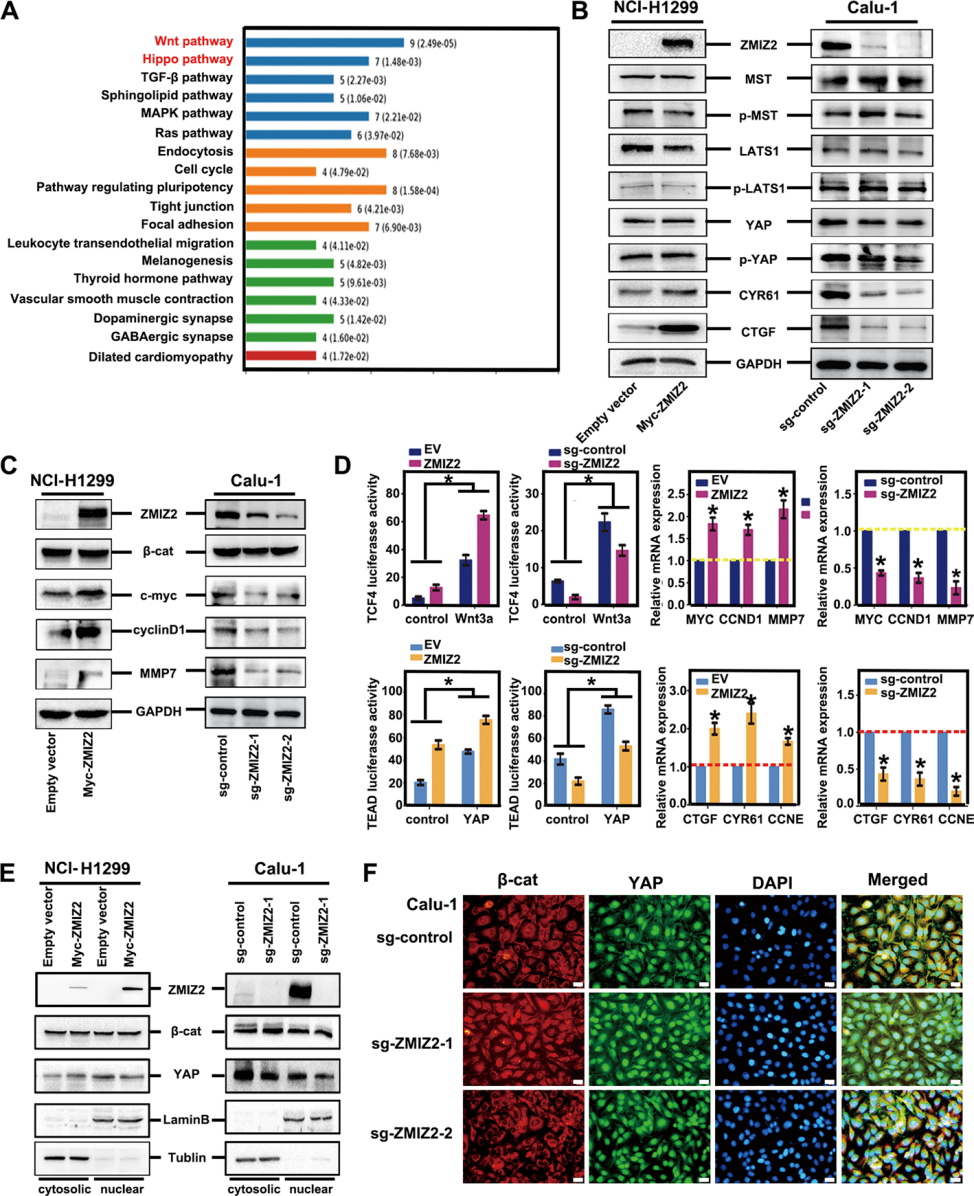
Role of ZMIZ2 as a regulator of the Wnt and Hippo signaling pathways. **A** Proteins identified through mass spectrometry analysis were subjected to KEGG analysis, revealing a close association between ZMIZ2 and the Wnt and Hippo pathways. **B**, **C** NCI-H1299 and Calu-1 cells were transfected with Myc-ZMIZ2 and sgRNA-ZMIZ2 plasmids, respectively. Western blotting was performed to assess changes in the key proteins related to Hippo (**B**) and Wnt (**C**) signaling, with GAPDH serving as a loading control. **D** NCI-H1299 and Calu-1 cells were cotransfected with ZMIZ2 plasmid and sgRNA-ZMIZ2, respectively, along with either Hippo pGL3b_8xGTIIC-luciferase or Wnt Super 8xTopflash plasmids. The cells were then mixed with the control, Wnt3A-, or YAP-conditioned media for 4 h. Renilla luciferase was used as a control for signal normalization. Luciferase reporter genes assays, and RT–qPCR were employed to examine the transcriptional activity of β-catenin-TCF4 and YAP–TEAD, as well as changes in the corresponding mRNA transcription levels of their target genes. Wnt3a (50 ng) and YAP amplification signaling proteins (2.5 μg) were included in the analysis. The relative transcript levels of genes were normalized to GAPDH mRNA levels. Data from a representative experiment are plotted as the mean of three replicates plus the standard deviation (*P < 0.05). **E**, **F** Cell nuclear–cytosolic protein separation experiment (**E**) and immunofluorescence (**F**, magnification, 400× ; scale bar, 50 μm) were performed to elucidate the impact of altering ZMIZ2 expression on the nuclear and cytoplasmic distribution of key factors, namely β-catenin, and YAP, in the Wnt and Hippo pathways. Tublin and LaminB1 served as cytosolic and nuclear loading control, respectively

We explored the molecular mechanism by which ZMIZ2 promotes the malignant phenotype of lung cancer cells. Utilizing bioinformatic methods (KEGG enrichment analysis from mass spectrometry), we identified and enriched a series of biological processes potentially linked to ZMIZ2. Our analysis revealed a close association between ZMIZ2 and the regulation of the Wnt and Hippo pathways (Fig. 4A). To validate this finding, we performed western blotting to detect the expression of key proteins associated with these pathways in cancer cells upon alteration of ZMIZ2 expression. Following ZMIZ2 transfection, significant upregulation was observed in the expression of key target genes of the Wnt pathway (*CCND1*, *MMP7*, and *C-myc*) and the Hippo pathway (*CTGF* and *CYR61*). Conversely, interference with ZMIZ2 expression yielded the opposite effect. However, the levels of the effector molecules in the two pathways, i.e., β-catenin and YAP, along with their upstream proteins, remained relatively unchanged (Fig. 4B, C). To validate these findings further, we performed luciferase reporter gene assay and RT–qPCR, which showed that ZMIZ2 overexpression significantly enhanced the transcriptional activity of TCF4 and TEAD, along with their downstream genes, mediated by β-catenin and YAP. Conversely, the knockdown of ZMIZ2 had the opposite effect (Fig. 4D). Simultaneously, we performed immunofluorescence and nuclear-cytosolic protein separation experiments, which revealed that bidirectional regulation of ZMIZ2 expression did not yet lead to a significant increase in the nuclear translocation of β-catenin and YAP (Fig. 4E, F). These results demonstrate that ZMIZ2 functions both as a positive regulator of the Wnt pathway and a negative regulator of the Hippo pathway. Moreover, these results indicate that the regulatory effects of ZMIZ2 on these pathways may not depend on classical upstream regulatory mechanisms.

### Combination of ZMIZ2 and SIRT1 promotes SIRT1’s deacetylation effect on β-catenin and YAP

**Fig. 5:**
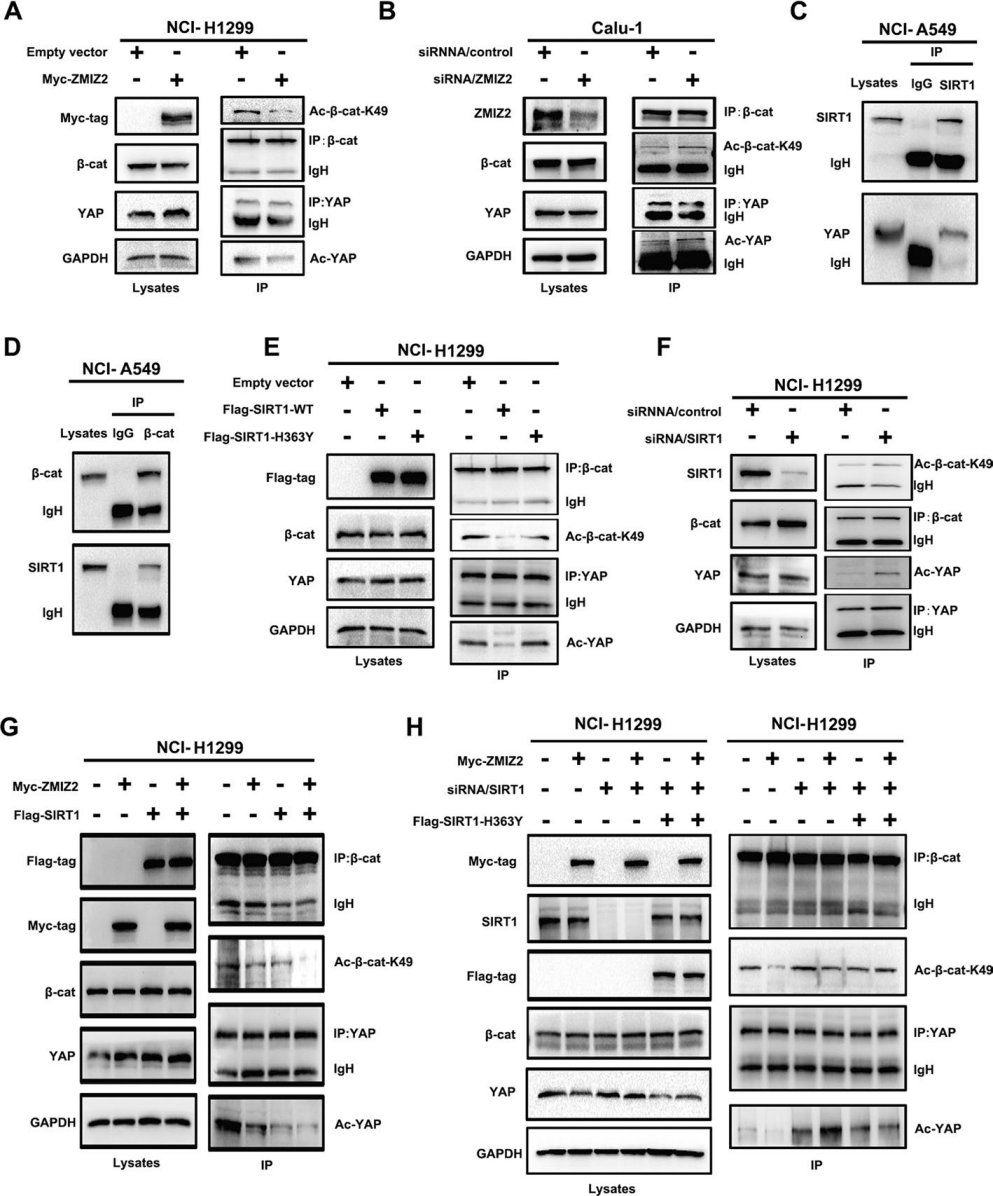
Impact of ZMIZ2 and SIRT1 combination on deacetylation of β-catenin and YAP. **A**, **B** NCI-H1299 and Calu-1 cells were transfected with ZMIZ2 and siRNA-ZMIZ2, respectively. Changes in β-catenin acetylation levels were assessed through immunoprecipitation and western blotting analysis. Total β-catenin and YAP levels served as loading controls. **C**, **D** Interaction between SIRT1 and β-catenin and YAP was validated using an endogenous immunoprecipitation assay in NCI-A549 and Calu-1 cells. **E**, **F** The impact of altering SIRT1 expression or deacetylase-deficiency (SIRT1-H363Y) on the acetylation levels of β-catenin (K49) and YAP proteins was assessed by immunoprecipitation assay. Total β-catenin and YAP were used as the loading control. **G** ZMIZ2 and SIRT1 plasmids were cotransfected into NCI-H1299 cell line, β-catenin (K49) and YAP protein acetylation were tested using immunoprecipitation assay. **H** Salvage experiments demonstrated that, in the absence of SIRT1 or with impaired SIRT1 acetylation function, ZMIZ2 could not enhance the downregulation of β-catenin (K49) and YAP protein acetylation levels. *IgH* IgG heavy chain

Previous studies have indicated that SIRT1, a nuclear deacetylase, facilitates the deacetylation of β-catenin (K49) and YAP, thereby promoting the transcriptional activity of the β-catenin-TCF4 and YAP–TEAD complexes [22, 23]. Building upon our earlier findings that ZMIZ2 interacts with the nucleus-localized deacetylase SIRT1 and is closely associated with the activity of Wnt and Hippo pathways, we contemplated whether ZMIZ2 influenced SIRT1’s deacetylase activity, thereby regulating the acetylation levels of β-catenin and YAP. To explore this hypothesis, we initially induced ZMIZ2 overexpression in the NCI-H1299 cell line, which resulted in a significant reduction in the acetylation levels of β-catenin (K49) and YAP. Conversely, the knockdown of ZMIZ2 in the Calu-1 cell line produced the opposite effect (Fig. 5A, B). In addition, coimmunoprecipitation and western blotting analyses conducted in the NCI-A549 cell line validated the interaction of SIRT1 with β-catenin and YAP in lung cancer cells (Fig. 5C, D). Furthermore, the overexpression of SIRT1 in NCI-H1299 cells downregulated the acetylation levels of β-catenin (K49) and YAP, whereas SIRT1-H363 (a deacetylation activity-deficient mutant) failed to abolish this effect (Fig. 5E). Conversely, the knockdown of SIRT1 resulted in the upregulation of both β-catenin (K49) and YAP acetylation levels (Fig. 5F). Furthermore, in NCI-H1299 cells, cotransfection of ZMIZ2 and SIRT1 plasmids led to a significantly enhanced regulatory impact on β-catenin (K49) and YAP deacetylation compared with the groups transfected with ZMIZ2 or SIRT1 alone (Fig. 5G). In contrast, in the recovery experiment, cotransfection of ZMIZ2 with siRNA-SIRT1 or SIRT1-H363 mutants revealed that ZMIZ2 did not exert significant effects on the deacetylation function of β-catenin (K49) and YAP in the absence of SIRT1 or when SIRT1’s deacetylase activity was deficient (Fig. 5H). Therefore, we concluded that ZMIZ2 enhances SIRT1's deacetylase activity by binding to SIRT1, thereby influencing the deacetylation of β-catenin and YAP.

### ZMIZ2 regulates the Wnt and Hippo pathway activities through SIRT1 and subsequently suppresses NSCLC malignant phenotype

**Fig. 6:**
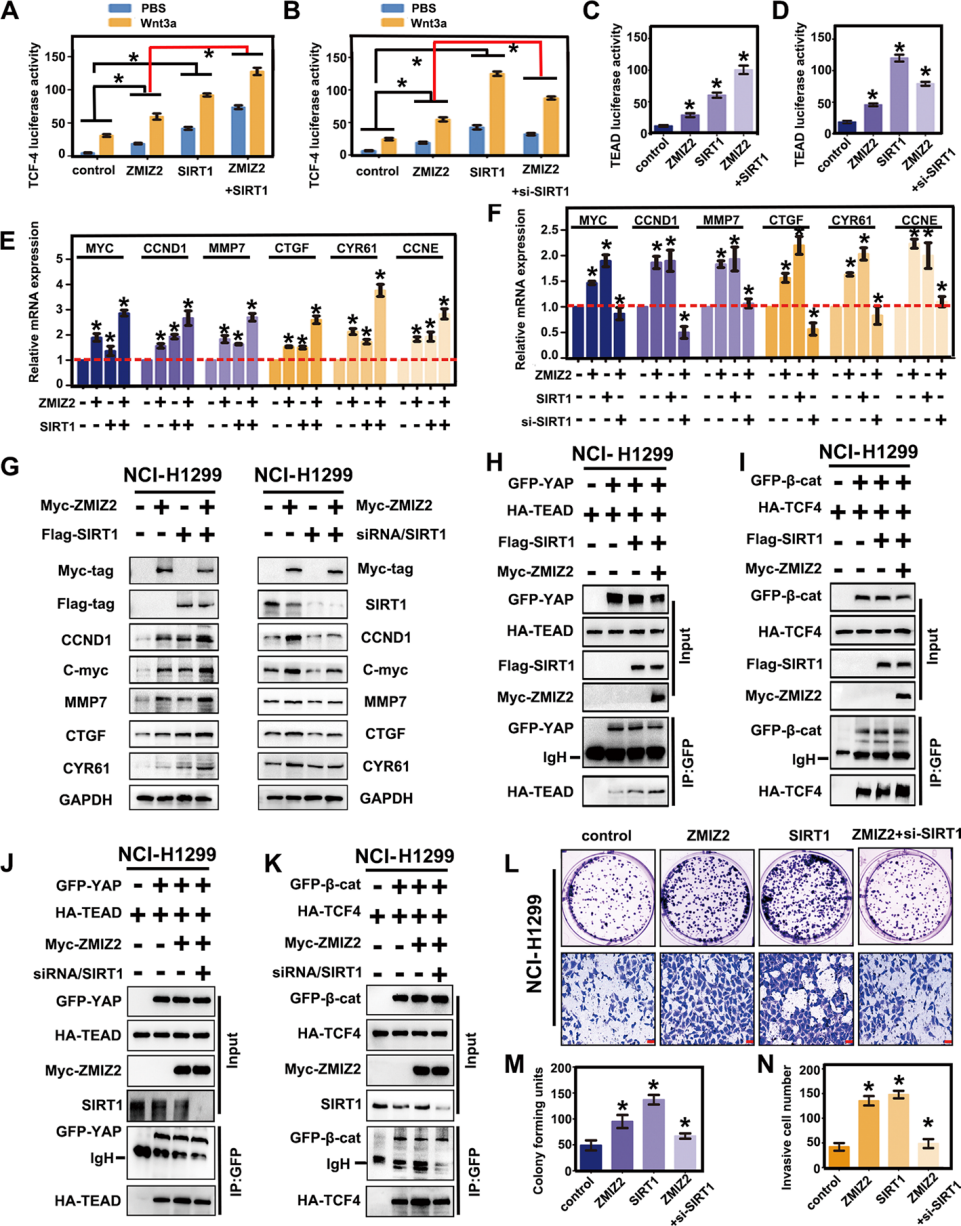
Role of ZMIZ2 in suppressing NSCLC malignant phenotype. **A–D** NCI-H1299 cells were transfected with ZMIZ2, SIRT1, and ZMIZ2 + SIRT1/siRNA-SIRT1, along with either Hippo pGL3b_8xGTIIC-luciferase or Wnt Super 8xTopflash plasmids. The cells were then mixed with control or Wnt3A-conditioned media for 4 h. Renilla luciferase was used as a control for signal normalization. Luciferase reporter gene assays were conducted to observe the changes in TCF4 (**A**, **B**) and TEAD (**C**, **D**) transcriptional activity. **E**–**G** RT–qPCR (**E**, **F**) and western blotting analyses (**G**) confirmed that ZMIZ2 upregulates mRNA and protein levels of Wnt and Hippo pathway target genes through SIRT1. Association with SIRT1 lead to significant reduction in the ZMIZ2-driven promotion of target gene expression. GAPDH served as a loading control. **H**, **I** Immunoprecipitation and western blotting assays indicated that cotransfection of ZMIZ2 and SIRT1 into NCI-H1299 cells led to ZMIZ2 synergistically promoting the enhanced binding of SIRT1-mediated β-catenin-TCF4 and YAP–TEAD complexes. **J**,** K** In the rescue experiment, the binding-enhancing effect mediated by ZMIZ2 on the β-catenin TCF4 and YAP–TEAD complexes disappeared after knocking down SIRT1. **L**,** M** Colony formation and Transwell experiments demonstrated that SIRT1 knockout substantially eliminated the enhanced malignant phenotype of lung cancer cells induced by ZMIZ2. Scale bar, 50 μm. The columns represent the mean numbers and the bars the standard deviation (SD); each experiment was performed triplicate. (*P < 0.05)

**Fig. 7:**
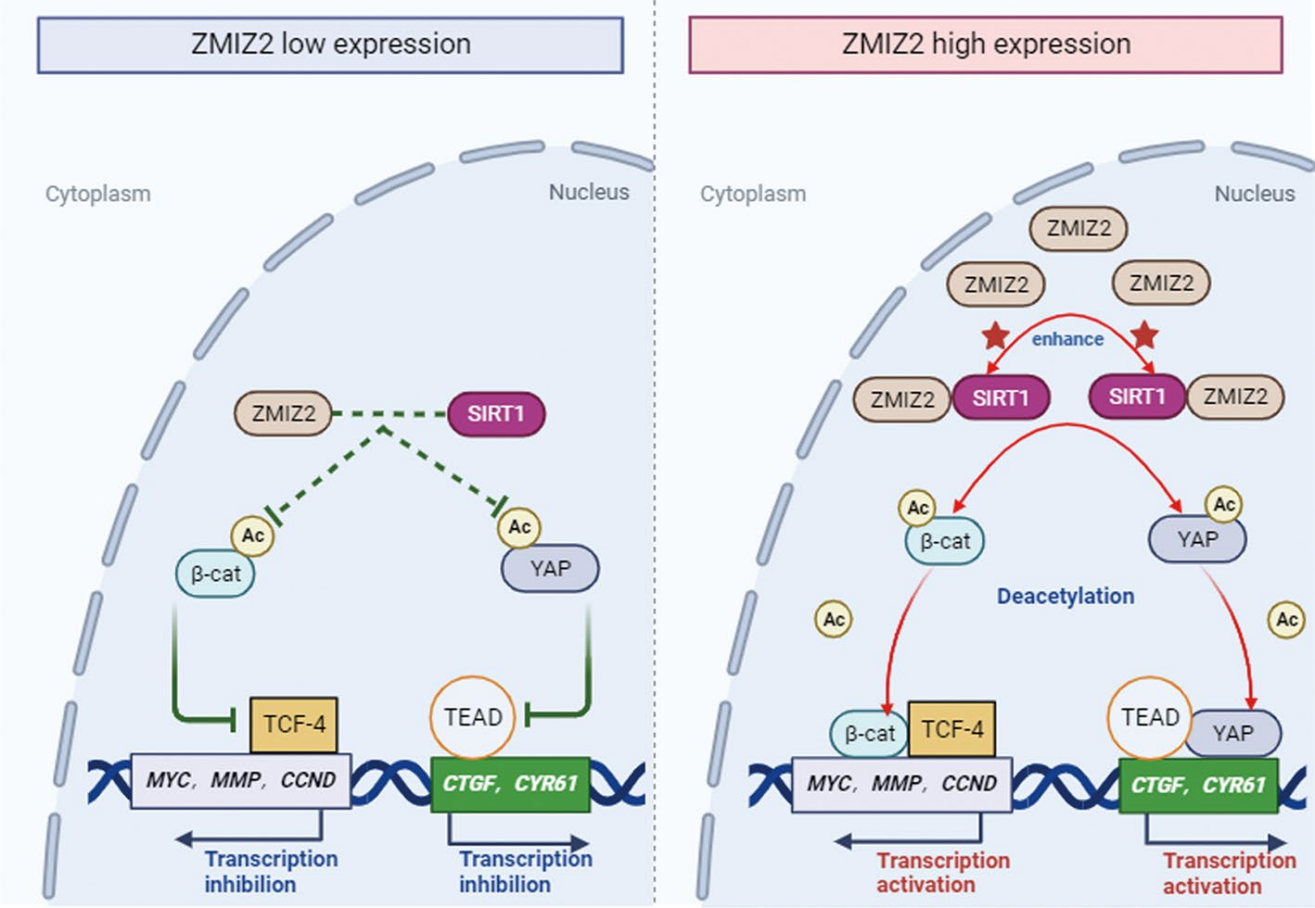
Schematic diagram of the molecular mechanism of ZMIZ2-mediated regulation of Wnt and Hippo signaling pathways

We demonstrated the combination of ZMIZ2 and SIRT1 plays a vital regulatory role in the post-translational modification of β-catenin and YAP. Consequently, we further examined the effect of ZMIZ2-SIRT1-mediated deacetylation on the cotranscriptional activity of β-catenin and YAP, as well as its effect on the malignant phenotype of lung cancer. The luciferase activity assay results showed that cotransfection of ZMIZ2 and SIRT1 in NCI-H1299 cells significantly increased the transcriptional activity of TCF4 and TEAD compared with the transfection with ZMIZ2 or SIRT1 alone (Fig. 6A, C). In contrast, SIRT1 knockdown abrogated the ZMIZ2-induced effects (Fig. 6B, D). Simultaneously, RT–qPCR and western blotting analyses demonstrated further upregulation of the expression of Wnt target genes (*CCND1*, *MMP7*, and *C-myc*) and Hippo pathway target genes (*CTGF*, *CYR61,* and *CCNE*) in the cotransfected group (Fig. 6E, G), while SIRT1 knockdown exerted the opposite result (Fig. 6F, G). To precisely elucidate the factors influencing the transcriptional activity of β-catenin and YAP and the changes in their target genes, we performed immunoprecipitation and western blotting analyses to reveal that cotransfection of ZMIZ2 and SIRT1 significantly enhanced the binding affinity between β-catenin and YAP with their corresponding transcription factors, TCF4 and TEAD (Fig. 6H, I). Conversely, SIRT1 knockdown abolished these effects (Fig. 6J, K). This suggests that ZMIZ2 enhances the activity of SIRT1-mediated cotranscription factors for β-catenin and YAP. Moreover, colony formation and transwell assays clearly demonstrated that siRNA-SIRT1 transfection significantly abolished ZMIZ2-induced enhanced proliferative and invasive capabilities of lung cancer cells compared with individual transfection (Fig. 6L–N). From these findings, we can conclusively assert that the interaction between ZMIZ2 and SIRT1 enhances SIRT1 activity, leading to the deacetylation of β-catenin/YAP. This process accelerates the formation of β-catenin-TCF4 and YAP–TEAD complexes, thereby activating Wnt and Hippo target gene transcription. Consequently, this dual activation of the Wnt pathway and inhibition of the Hippo pathway contribute to the promotion of the malignant phenotype in NSCLC (Fig. 7).

## Discussion

Lung cancer is the most prevalent among all malignant tumous, considering its high incidence and mortality rates. NSCLC, including adenocarcinoma, squamous-cell carcinoma, and large-cell carcinoma account for approximately 85% of overall lung cancers. Although significant advancements have been achieved in the fields of surgery, chemotherapy, and targeted therapy, most patients continue to experience a poor prognosis owing to late-stage diagnosis and reduced drug sensitivity following surgery [24, 25]. The revelation of the molecular mechanisms governing the invasion and metastasis of lung cancer, as well as the identification of novel biomarkers and therapeutic targets for its biological behavior hold substantial value in enhancing the prognostic assessment of lung cancer.

Our study revealed elevated ZMIZ2 expression in various lung cancer cell lines and NSCLC cases. Functional assays conducted post-transfection with ZMIZ2 or ZMIZ2 sgRNA plasmids showed that ZMIZ2 might promote the proliferation and invasion capabilities of lung cancer cells. These findings indicate ZMIZ2 plays a significant role in the malignant progression of lung cancer, consistent with the findings of previous studies on colon, triple-negative breast cancers, and hepatocellular carcinoma, underscoring the oncogenic role of ZMIZ2 across various malignancies [15–17]. Besides, ZMIZ2 was screened to be the target gene regulated by the IGF2BP3/circRARS complex in an m^6^A-dependent manner in renal carcinoma [26]. While our study substantiates the heightened ZMIZ2 expression in lung cancer tissues and cell lines compared with that of normal lung tissues, the specific mechanisms driving this upregulation remain unclear. Future investigations are warranted to discern whether this increase occurs at the transcriptional level (involving factors such as promoter amplification, transcription factors regulation, or noncoding RNA) or at the post-transcriptional protein expression level.

In our investigation of the molecular mechanism by which ZMIZ2 promotes the malignant phenotype of lung cancer cells, we found that ZMIZ2 significantly enhanced the expression of the key target genes associated with the Wnt pathway. These findings were substantiated by luciferase reporter gene assays, which demonstrated ZMIZ2’s capacity to promote Wnt pathway activity. Immunoprecipitation experiments validated the interaction between ZMIZ2 and β-catenin, aligning with earlier findings reported by Lee et al. [14] in prostate cancer cells. These results also corroborate the observed interaction between ZMIZ2 and SIRT1. Notably, following ZMIZ2 transfection, we observed a substantial increase in the binding of β-catenin to TCF4, which implies that the upregulation of the acetylation of β-catenin could enhance the affinity between β-catenin and TCF4. This novel insight elucidates the specific molecular mechanism by which ZMIZ2 activates Wnt pathway.

However, the question remains: Why does ZMIZ2 transfection enhance the binding of β-catenin to TCF4? Existing literature suggests that the deacetylase SIRT1 can bind with β-catenin, leading to the deacetylation of its 49th lysine site. This process, in turn, promotes the transcriptional activity of the β-catenin-TCF4 complex, ultimately activating the Wnt pathway [20]. Another noteworthy finding is the interaction between ZMIZ2 and the deacetylase SIRT1 in the nucleus. Consequently, ZMIZ2 overexpression resulted in a significant downregulation of β-catenin acetylation. Our findings provide a basis for elucidating the specific mechanism through which ZMIZ2 influences Wnt pathway activity. We demonstrate that ZMIZ2 initially forms a complex with SIRT1. This interaction subsequently facilitates the binding of SIRT1 to β-catenin, leading to its deacetylation. Consequently, this process promotes the formation and transcriptional activity of the β-catenin-TCF4 complex, thereby activating the Wnt pathway.

Notably, there remains a perplexing question and a flaw in our research. In our exploration of the protein domains that are crucial for the interaction between ZMIZ2 and SIRT1, we solely modified the well-known domains of ZMIZ2. However, we discovered that ZMIZ2 cannot bind to SIRT1 unless the NLS sequence is present. This suggests that the binding of ZMIZ2 to SIRT1 may rely on other regions beyond the three known domains, such as the SP-RING domain in ZMIZ2, which are involved in sumoylation. In addition, it is worth to note that ZMIZ proteins have been shown to interact directly with transcription factors (TFs), as is the case for ZMIZ1 in the stimulation of NOTCH-mediated transcription during T-cell development and leukemia [27, 28]. We cannot rule out a direct interaction of ZMIZ2 with other unknown TFs to stimulate transcription that could regulate the Wnt or Hippo pathways, which need further investigation for us.

Broad crosstalk exists between the Wnt and Hippo pathways [29–32]; thus, we also explored the potential impact of ZMIZ2 on the Hippo pathway. The luciferase reporter gene experiment showed that ZMIZ2 not only modulates the Wnt pathway but also influences Hippo pathway activity. Identifying the crucial intersection between these two pathways was a key focus of our investigation. Our literature review revealed that SIRT1 potentially interacts with YAP, deacetylating the YAP lysine site [22]. This interaction enhances the binding of YAP to TEAD, consequently inhibiting the Hippo pathway. Moreover, our findings indicate that the interaction between ZMIZ2 and SIRT1 upregulates the activity of SIRT1, which plays a dual role; initially, it induces the deacetylation of β-catenin, thereby accelerating the formation of the β-catenin-TCF4 complex and facilitating the transcription of Wnt target genes. Simultaneously, the interaction between SIRT1 and YAP results in YAP deacetylation. This enhances the binding affinity between YAP and TEAD, ultimately downregulating the transcriptional activity of the Hippo pathway.

## Conclusions

The interaction between ZMIZ2 and SIRT1 results in the activation of the Wnt pathway and inhibition of the Hippo pathway, thereby promoting the biological phenotypes of lung cancer, including cell proliferation, invasion, and metastasis. These research findings not only elucidate the role of ZMIZ2 in promoting lung cancer progression through the Wnt and Hippo pathways but also suggest potential avenues for personalized treatments.

## Supplementary Information

Supplementary Material 1

## Data Availability

All data generated or analyzed during this study are included in this published article and its additional files. Further details were available from the corresponding author upon request.

## Abbreviations

ZMIZ2: Zinc finger MIZ-type containing 2
SIRT1: NAD-dependent protein deacetylase sirtuin-1
LATS: Large tumor suppressor
MST: Mammalian sterile-20-like
YAP: Yes-associated protein
CTGF: Connective tissue growth factor
CYR61: Cysteine-rich angiogenic inducer 61
MMP7: Matrix metallopeptidase-7
RT–qPCR: Reverse transcription-quantitative polymerase chain reaction
GAPDH: Glyceraldehyde-3-phosphate dehydrogenase
TCF4: Transcription factor 4
TEAD: TEA domain family protein
IgH: Immunoglobulin heavy chain
sgRNA: Small guide RNA
NSCLC: Non-small-cell lung cancer
KEGG: Kyoto Encyclopedia of Genes and Genomes
PBS: Phosphate-buffered saline
NSCLC: Non-small-cell lung cancer
IHC: Immunohistochemistry
NLS: Nuclear localization sequence
IP: Immunoprecipitation
IB: Immunoblotting
